# Remaining caries-free – a personal concern: an interview study to capture the impact of salutogenic influencing factors in caries-free middle-aged adults

**DOI:** 10.1186/s12903-026-08350-2

**Published:** 2026-04-17

**Authors:** Cajsa Fabricius, Adam Alvenfors, Peter Lingström, Annica Almståhl, Jenny Bernson

**Affiliations:** 1 Department of Cariology, Institute of Odontology, Sahlgrenska Academy, University of Gothenburg, Box 450, Gothenburg, SE-405 30 Sweden; 2 Research, Education, Development & Innovation, Public Dental Service in Region Västra Götaland, Gothenburg, Sweden; 3 Department of Oral Microbiology and Immunology, Institute of Odontology, Sahlgrenska Academy, University of Gothenburg, Gothenburg, Sweden; 4 Department of Oral Health, Faculty of Odontology, Malmö University, Malmö, Sweden

**Keywords:** Adults, Caries, Caries-free, Dental health factors, Salutogenic

## Abstract

**Background:**

Caries is considered the most prevalent disease worldwide. The prevailing paradigm in caries research and care has historically focused on the identification and management of high-risk patients and the disease itself. In recent years, the salutogenic perspective has received increased attention. This perspective has the potential to offer novel insights into the mechanisms by which the disease may be treated. This study aimed to capture experiences related to the impact and use of salutogenic factors influencing the maintenance of a caries-free status among adult individuals.

**Materials and methods:**

To ensure credibility and data quality, caries-free participants were strategically recruited through purposive sampling via an aggregated database of dental records. The study sample included 15 adult individuals (> 40 years), eight males and seven females from diverse socioeconomic backgrounds. Data were collected through semi-structured in-depth interviews. The interviews were audio-recorded and transcribed verbatim, after which they were analyzed using the qualitative content analysis method, with an inductive approach.

**Results:**

The overarching theme identified *‘Remaining caries-free – a personal concern’*, reflects the participants’ proactive engagement in their own oral health, and included a consequentialist mindset. Six key themes emerged: ‘External impact’, ‘Assisted encouragement’, ‘Acquired knowledge’, ‘Protective characteristics’, ‘Positive habits’, and ‘Personal commitment’. These influencing themes emerged from three more descriptive subthemes each, which formed the basis and provided deeper insight into the theme’s more broader contents. The results underscore the importance of a supportive social environment in fostering oral health-promoting habits, highlighting the importance of receiving support and guidance from family, friends, and society during childhood and adolescence. Furthermore, the results indicate that a meticulous personality and a well-informed approach to health may be crucial factors for maintaining a caries-free status in adulthood.

**Conclusion:**

A deeper understanding of the salutogenic influences that contribute to long-term caries-free status, including both the formation and maintenance of good habits from childhood to adulthood, could guide individual empowerment of self-efficacy and enhance the effectiveness of prevention strategies in dental care. This underscores the need for further exploration in this area from a lifeworld perspective where care should be person-centered also for those free of caries.

## Background

It is estimated that more than 2 billion people of all ages worldwide are affected by untreated dental caries, a disease with a well-documented etiology [1–3]. In middle- and low-income countries, the prevalence of dental caries is high, whereas in high-income countries, the prevalence has declined dramatically since the middle of the last century, and the current caries rate is among the lowest in the world in modern times. However, the decline has levelled off, and the prevalence is now relatively constant [1, 2, 4].

In Sweden, there was a marked decline in caries prevalence among 40-year-olds between 1973 and 2013, and the number of caries-free 15-year-olds increased significantly over the same period [5]. A more recent study revealed a slight decline in DFS in adults aged 40–70 years [6]. Despite this, recurrent caries persists in parts of the population, and current, management strategies have not been sufficient to achieve disease inactivity.

In the absence of adequate caries control measures, there is a risk of adverse consequences for both oral health and general health, as well as quality of life [7]. The financial burden associated with dental caries is significant, affecting both the individual and society at large [8].

Considering the significant and complex impact of caries on both individual well-being and societal costs, it is essential to investigate not only the risk factors contributing to the development of caries disease but also the protective factors that can prevent the disease from occurring. Traditionally, identifying individuals at risk of developing caries and those with active caries has been the focus of caries management in dentistry [9]. In recent years, the salutogenic perspective has been highlighted in caries research [10]. Antonovsky [11] defines salutogenesis as a system theory where the coherence between the individual, the community, and the environment plays an important role in the development of the sense of coherence (SOC). SOC is a measure of an individual’s ability to maintain health despite difficulties in life. Salutogenic factors are defined as the elements that strengthen an individual’s SOC and promote health by facilitating adaptation and the management of challenges in life. SOC is relatively well discussed in the context of caries [12]. However, being caries-free cannot be explained simply by having a high SOC.

In health and care sciences, a salutogenic perspective on research means focusing on opportunities and reasons for individuals to remain healthy. Previous dental research with a salutogenic perspective has focused on the broader concept of ‘oral health’ and has included mainly children and adolescents [10, 13, 14] but not adults. Despite growing interest in health-promoting approaches to caries prevention, the existing literature remains dominated by studies focused on pediatric and adolescent populations and predominantly employs a pathogenic rather than a salutogenic perspective. Research exploring why some individuals remain caries-free into middle and older age is notably scarce — a gap that is partly attributable to the low prevalence of such individuals, and partly to the terminological heterogeneity of the field, where related concepts such as resilience, coping, and health literacy are often explored in isolation rather than within a unified salutogenic framework. To the best of our knowledge, no previous study using a qualitative method has explored caries-free adults’ lived experiences and perceived health-promoting factors from a salutogenic perspective. This study therefore aims to address this knowledge gap.

Current strategies to prevent dental caries are predicated on the disease itself and encompass direct disease control measures [15]. Understanding why some individuals develop caries while others do not is important when trying to understand salutogenic caries-preventing factors. A more effective preventive strategy would result from a better understanding of the factors contributing to remaining caries-free in the long term. The integration of a salutogenic perspective could provide novel insights or a more comprehensive understanding of the factors that govern remaining caries-free throughout life, and new insights into caries preventive strategies. Resources within dental care could be reallocated, which could lead to additional and potentially improved treatment methods for individuals with caries activity. The aim of this interview study was to capture experiences related to the impact and use of salutogenic factors influencing the maintenance of a caries-free status among adult individuals.

## Materials and methods

### Study design

A qualitative research approach was used to obtain a deeper understanding and knowledge regarding experiences and perceptions of salutogenic factors impact among caries-free adults. The study relies on qualitative content analysis with an inductive approach, in accordance with Graneheim and Lundman, a well-established and widely used method across various research fields, particularly in health care [16]. Qualitative content analysis allows researchers to systematically explore and interpret both the manifest and latent meaning of human experiences and to draw further conclusions about the content of the data at different levels of abstraction and interpretation [17]. Given its suitability for studying human experiences, this method was deemed appropriate for the study.

### Ethics

The study was conducted in accordance with the World Medical Association Declaration of Helsinki and was approved by the Swedish Ethical Review Authority (reference number 2020–04819). The privacy rights of human subjects were observed, and the participants were, prior to the data collection, provided with both verbal and written information regarding the objectives of the study and the possibility of withdrawing. Written informed consent was obtained.

### Recruitment of informants

The informants in the study consisted of adults from different parts of Region Västra Götaland in Sweden. The inclusion criteria were patients listed at the Public Dental Service in the region, aged 40 years or older, and with caries-free permanent dentition according to dental records (i.e., a DFT of 0). The exclusion criterion was poor ability to understand the Swedish language. Fifteen informants, 8 men and 7 women aged 41–63 years, were recruited. In accordance with the inclusion criteria, the selection of interviewees was initially performed via an aggregated database of dental records (T4 in Public Dental Service, Region Västra Götaland). As the data collection progressed, the selection of informants became more strategic and theoretical to ensure high quality of data. This refinement allowed for the inclusion of a more diverse sample, as it included informants with different experiences from diverse locations in the region, from rural areas to larger cities, to ensure the quality of the data and refine the emerging theoretical framework [18].

### Data collection

The study comprised 15 interviews conducted by the two authors, AA^a^ and CF. AA^a^ had prior experience in performing research interviews and therefore conducted the initial two interviews with CF present as an observer. CF subsequently conducted the remaining thirteen interviews, with AA^a^ present during the initial ones to ensure quality and consistency. JB acted as supervisor for the generation and transcription of data. Owing to the prevailing circumstances of the COVID-19 pandemic, the interviews were held via the digital meeting tool Zoom. The interviews were conducted and audio-recorded from November 2021 to April 2023. The interviews lasted between 33 and 63 min.

Data were collected through semi structured in-depth interviews with some preconstructed and pilot-tested questions as interview guidance [19]. The pilot involved testing the interview questions on individuals within the research group’s network, which also provided an opportunity to refine the guide and train the interviewer. During the actual data collection, the guide was revised to serve only as a support to follow up questions to broaden and deepen the informants’ communication of experiences.

The interviews began with sociodemographic questions about age and residence during child- and adulthood. The subjects were then given open-ended inquiries regarding food intake, oral care, and fluoride use. Furthermore, the subjects were given open-ended inquiries regarding their experiences of remaining caries-free and the factors they believed had contributed to their sustained caries-free status into adulthood, and the strategies used to remain caries-free. Examples of questions asked:

‘I am interested in hearing your thoughts on what factors you believe have contributed to you remaining caries-free for so long’.

‘Is there anyone in your family or in your immediate vicinity who has influenced you positively and if so, in what way?’

‘Tell me about your previous experiences with dental care and how these could have influenced your avoidance of caries.’

‘Describe how you take care of your mouth.’

The interviews were audio-recorded and transcribed verbatim. After 13 interviews, the data collected was considered rich and varied. A decision was made to conduct two additional interviews in an attempt to obtain data from broader perspectives. As these interviews did not generate any new information or insights, the decision was made to end the data collection at 15 interviews. Prior to analysis, both the recordings and transcripts were deidentified and coded.

Researchers involved in data collection and analysis must understand the research process and have some prior understanding of the research area, to guide interviews towards relevant data and delimit the area of ​​interest of the research question. This requires both creativity and curiosity, as well as the ability to change perspectives. Furthermore, a theoretical sensitivity and reflection of own pre-understanding are important in the collection and analysis process [20].

### Data analysis

The inductive qualitative content analysis, of the data was conducted collaboratively by all researchers involved in the study [18, 21]. The analysis comprised several stages. Initially, the researchers read the transcribed interview texts individually and formed an overall impression of their content. The text was divided into meaning units and further condensed and labeled with codes in accordance with the established methodology. The codes were categorized into descriptive themes and category themes. These themes were finally abstracted and interpreted into a main theme. Disagreements during the analysis process were resolved through discussions between the researchers to find consensus.

During the interviews and analysis, the researchers adopted an unprejudiced approach to interpret the data with the goal of not being influenced by their own theories. Therefore, the researchers reflected on and managed their preunderstandings [22]. The researcher analyze group consisted of; CF, a female DDS/PhD student, who has significant clinical experience in public dental care and specialist cariology training, and AA^a^, a male DDS/PhD student with an additional degree in psychology, who has experience working as a general dentist in rural Sweden. PL, a male dentist and professor, who has significant clinical and research cariology experience. AA^b, c^ a female dental hygienist and associate professor in oral health science, with some experience working as a dental hygienist in public dental care, and JB, a female DDS and researcher with a PhD in odontology, who has extensive experience treating dental anxiety and dental research via qualitative methodologies. It could also be mentioned that one of the interviewers and two of the totally five authors were caries-free, with no previous experience of caries themselves.

## Results

### The resulting picture

The results revealed a main theme, ‘Remaining caries-free—a personal concern’. In addition, six underlying influencing themes were identified: External impact, Assisted encouragement, Acquired knowledge, Protective characteristics, Good habits, and Personal commitment. These influencing themes reflect the informants’ views and experiences of why they had remained caries-free into adulthood. This framework of themes formed a pattern of underlying more descriptive subthemes (Table 1) and the content of the subthemes, all based on the collected data, was further reported in the results, illustrated with quotations from the informants.

**Table 1:** The main theme, influencing themes, and descriptive subthemes that emerged from the qualitative content analysis of data

Remaining caries-free – a personal concern Where good oral health was highly valued and oral problems managed using a consequentialist mindset	
*Influencing themes*	*Descriptive subthemes*
EXTERNAL IMPACT	• Family: experiences, morals, and personal openness • Other individuals: knowledge, advice, and opinions • Society: information, attitudes, and messages
ASSISTED ENCOURAGEMENT	• Lifestyle influences and assisted toothbrushing at home • Fluoride administration, oral hygiene instruction, and nutritional information in school • Dietary information and oral hygiene instruction in children’s dental care
ACQUIRED KNOWLEDGE	• Search for information • Acknowledgment of caries as a disease • Reflection on reasons for being caries-free
PROTECTIVE CHARACTERISTICS	• Strong teeth • Low craving for sugar • Good secretion and composition of saliva
GOOD HABITS	• Limited number of meals per day and low intake of sugar • Good oral hygiene routines • Regular use of fluoride products
PERSONAL COMMITMENT	• Maintenance of good routines important for oral health and regular dental care • Compliance with advice and instructions • Expressions of benefits and gratitude for remaining caries-free

### The main theme: remaining caries-free – a personal concern

The entire result was permeated by the informants’ expressions that they considered the mouth to be an important part of the body and therefore made it well worth the effort to care for. The subjects expressed appreciation for the freshness and naturalness of the mouth where they wanted to avoid aesthetic and cosmetic dentistry, indicating a preference for a holistic approach to dentistry. They conveyed a belief that good oral health is indicative of and influences general health.

The ability to remain caries-free emerged as a personal concern during youth, gradually solidifying into a core foundation as the informants transitioned into middle age. Over time, this concern deepened, evolving into a dynamic and enduring mindset. The use of a consequentialist approach guided preventive and deliberate actions to maintain oral health. Building on this foundation, the informants developed a consequentialist mindset that motivated them to take timely action to avoid oral problems and prevent unnecessary, extensive, troublesome, and costly dental treatments. The informants also appreciated the support and help they had received from society, family, and friends. They valued the good patient care, regular visits, and preventive efforts that dental professionals in public dental care had provided. Finally, they expressed that their personal commitment and efforts to remain caries-free were important and, in turn, made them feel proud.

### Influencing themes and descriptive subthemes

#### External impact

- Family: experiences, morals, and personal openness
- Other individuals: knowledge, advice, and opinions
- Society: information, attitudes, and messages

The informants stated that during childhood, they already had knowledge of the oral health conditions of their family members, which could be seen as a sign of openness about this personal and revealing subject. There was also a willingness among older family members to transfer personal experiences and their own moral aspects regarding oral health so that the children would be able to have better oral health than themselves:

*(I8) “Both mum and dad had amalgam fillings. Mum removed them and replaced them*,* maybe 20 years ago. That was when it was a bit popular to do so. My brother does not have any cavities as far as I know*,* I don't think so*,* and my uncle has had very bad teeth. He has many [teeth] that are not real.”*

Furthermore, the informants were continually influenced by knowledge, advice, and attitudes from others such as friends and acquaintances:

*(I3) “My mum’s friend ‘M’*,* who is a dental nurse*,* has always been this careful with us too. Ehh*,* so I know that when we were little*,* and we were there*,* then it could be like that: ‘You can let ‘M’ brush your teeth’. Good*,* and then you had to stand there*,* and she brushed.”*

Experiences were shared on the impact of attitudes and societal culture and on impressions, both positive and negative, from different public advertisements regarding white, healthy, and successful smiles.

*(I4) “You cannot separate the head from the rest of the body*,* so to speak. It's is all connected*,* and that's really obvious... so I’m even more aware of dental care now than I was before.”*

Some informants noted that information from the state and municipality via various institutions, such as schools, the health care system, and media, helped them develop attitudes about oral hygiene:

*(I6) “You should simply brush your teeth and take care of them. I think it is deeply ingrained in Swedish society.”*

#### Assisted encouragement

- Lifestyle influences and assisted toothbrushing at home
- Fluoride administration, oral hygiene instruction, and nutritional information in school
- Dietary information and oral hygiene instruction in children’s dental care

The informants stated that they had been brought up to eat nutritious food at home, so they did not have to snack between the meals. They were accustomed to drinking milk or water, and soda and juice were only available at parties or in some homes on weekends. They had also been brought up to eat sweets only on Saturdays, ‘Saturday sweets’, which had become established in the 1950s in Sweden.

*(I10) “When I was growing up*,* I did not eat very many sweets except maybe on Saturdays and never drank a lot of juice and soft drinks and things like that.”*

As children, the informants were encouraged not to neglect their oral hygiene, and they received help with brushing their teeth, often even after they reached school age, from their parents or other relatives.

*(I7) “We have had parents who have been very strict with us about brushing our teeth in the morning and evening and not eating after brushing our teeth.”*

In the 1970s and 1980s, the prevalence of caries in children in Sweden was high. Therefore, regular visits from a dental nurse—‘The fluoride-aunt’ (a direct translation from Swedish)—were introduced in schools. The dental nurse taught the children about their dental health and unhealthy food and provided fluoride rinses. The informants reported that fluoride rinses were positive and that something that they thought helped them to remain caries free. However, even if the informants rinsed, their schoolmates were not always positive, as they thought the fluid had an unpleasant taste and therefore tried to avoid it.

*(I2) “At least I was one of those who*,* when the fluoride-aunt was in the corridor*,* I took it*,* while many others walked by and complained that it tasted so disgusting.”*

The informants had good experiences going to dentistry, both as children and as adults. Some described that it could feel a little uncomfortable because it was a sensitive or intimate procedure; others reported that they felt no problems. The visits were described as pleasant with good patient care. During these visits, they received dietary information and instruction in dental hygiene. Many had also received polishing and fluoride varnishing of their teeth.

*(I10) “Yes*,* but I have always liked going to the dentist; there have never been any problems. It is only good experiences and that you are called for examination and so on*,* yes*,* with a friendly approach.”*

#### Acquired knowledge

- Search for information
- Acknowledgment of caries as a disease
- Reflection on reasons for being caries-free

The informants obtained information and knowledge of dental diseases in different ways, for example, through oral transmission during dental visits as well as through an active search for information and knowledge in scientific journals or via the internet.

*(I4) “More recently*,* you may have read a bit more on the internet. Not just random articles like that*,* but I have looked for reliable sources about what’s the most up-to-date information.”*

The informants had a high level of knowledge about caries and its etiology in terms of the negative effects of bacteria, sugar, and snacking on dental health.

*(I2) “Caries are bacteria that thrive in sugar and plaque and then destroy the enamel.”*

They also suggested that there could be a genetic link to the development of caries. Interestingly, the informants perceived differences between caries and erosion and other forms of tooth wear, as well as perceptions of the causes of gingivitis and periodontitis and how oral conditions could affect the mouth and, to some extent, general health.

*(I6)* “*And that should not be confused with the corrosive damage that you can get from soft drinks and juices and such*,* because then it is more like the acids*,* quite strong acids that are present.”*

*(I4) “Therefore*,* I have become a little pickier actually*,* with dental care... I have understood that dental care is very important not only for the teeth but also that it affects the rest of the body with various types of inflammation and other diseases*,* and I have learned that lately.”*

In addition, the informants reflected on why they had remained caries-free and what they thought had led to this. They had many thoughts about the reasons for their healthy teeth in comparison to what could have caused caries in others whom they knew.

*(I11) “I actually don’t know why *[*I’m caries-free*], *maybe it’s just that I’m careful. I brush my teeth as I should do every day and floss my teeth and then... I can’t say that I’m one of those people who doesn’t eat sugar or something because I do.”*

#### Protective characteristics

- Strong teeth
- Low craving for sugar
- Good secretion and composition of saliva

During the interviews, the informants tried to summarize or embrace the individual factors that they believed could explain why they had not been affected by caries. These individuals are believed to have good genetics and strong teeth.

*(I2) “My dental health is probably due to good genetics*,* whatever that is... combined with the fact that I have taken care of myself from childhood.”*

*(I15) “Both my twin sister and I are caries-free*,* so there is probably a genetic explanation.”*

At the same time, they expressed amazement at remaining caries free and conveyed that they were both proud of and grateful for their healthy teeth.

*(I6) “It is just that I’m still just as surprised that I have never had a cavity because I do the same things that many*,* many others do. And their oral health is getting worse*,* but mine is not. I simply do not understand.”*

Another factor they emphasized was their low intake of sugar and sweets, which was explained by the perception that they did not feel the same sugar cravings as many others do, which was partly addressed to genetics. As well as sugar cravings being genetic, good eating habits are also suggested to be an important part of taking care of oneself.

*(I3) “My husband has had many more cavities and has fixed several [teeth]. However*,* then I also know that he eats a lot more sweets and has a preference for it.”*

Saliva composition and good saliva secretion were also highlighted as important factors that, although not considered decisive, could affect oral health.

*(I14) “The mouth tries to clean itself*,* depending on how effective it is in the mouth*,* the more saliva you have*,* the faster it goes. However*,* if it never gets to finish its job*,* it more or less corrodes all the time.”*

#### Good habits

- Limited number of meals per day and low intake of sugar
- Good oral hygiene routines
- Regular use of fluoride products

Since childhood, the informants were accustomed to eating various healthy foods regularly and not snacking between meals or consuming junk food. They were also accustomed to not consuming sweets daily or drinking sweetened beverages, and they maintained these habits into adulthood.

*(I3) “I don’t think I have snack between meals either*,* but I eat quite a lot when I eat*,* and I eat regularly. It’s a proper breakfast*,* lunch*,* and dinner always. Uh*,* so that could also contribute to resting the teeth.”*

*(*I6*) “We definitely ate sweets on Saturdays*,* but we never drank soft drinks or anything like that*,* still don't.”*

They stated that the habit of not eating sweets randomly was a crucial factor and distinguished them from the people they knew had caries. Furthermore, the subjects reported that they believed it was significantly more difficult to establish and maintain good habits in today’s society, which they attributed to the greatly increased availability of sweetened beverages and nutrient-poor foods.

The informants described themselves as people who maintained a routine, rarely forgetting to brush their teeth even when they were tired. They also brushed their teeth as a way to unwind and prepare for sleep.

*(I10) “No matter how tired you are*,* you always stick that toothbrush in your mouth.”*

*(I6) “In the evening as a little way to unwind*,* I can probably brush for five*,* six*,* seven minutes sometimes if I can walk around and philosophize as I like with the toothbrush in my mouth. So*,* it is quite a nice moment like that.”*

The informants agreed that the use of fluoride was important for dental health and that they used fluoride toothpaste. Some also used various forms of fluoride supplements, and they were very knowledgeable about the fluoride content of the products.

*(I4) “In regard to toothpaste*,* I mainly look at the fluoride content. I have always chosen those with the highest amount of fluoride*,* 1450 ppm*,* which is the highest you can obtain*,* at least in the department stores.”*

They were certain that the use of fluoride had benefitted their dental health, but they did not believe that it could compensate for dietary mismanagement.

From childhood, the informants had carried these habits and routines with them into adulthood. They also encouraged their children to adopt these routines because they believed that good habits and routines from the beginning were crucial for a caries-free life and for maintaining good oral health.

*(I7) ”It costs an awful lot of money; I know it does. However*,* yes*,* you can think about it now for the next generation*,* so that you make sure that he takes care of his teeth*,* because it can be expensive for you when you get older otherwise.”*

#### Personal commitment

- Maintenance of good routines important for oral health and regular dental care
- Compliance with advice and instructions
- Expressions of benefits and gratitude for remaining caries-free

The respondents expressed that the most important factor in achieving optimal dental health was committing to daily oral care and developing sound habits and routines. Their commitment to dental health was reflected in their attention to diet and oral hygiene, including the use of fluoride. A common reason given by the respondents for their diligence in caring for their teeth was that they were meticulous and conscientious in all aspects of life.

*(I2) “I don't think it's specifically the teeth I have been careful with; I have been thorough with everything I have done. I was careful with my toys*,* I was careful with my teeth*,* and I was careful with*,* well*,* most things.”*

Furthermore, the importance of attending the check-ups offered by the dental clinic was emphasized, as was the importance of accepting the support provided and adhering to the information and instructions issued by the dental professionals.

*(I4) “You reap what you sow; if you take care of your dental health according to the dentist’s recommendations*,* you don’t have to get cavities and many problems.”*

The ability to remain caries-free was experienced as an advantage that encouraged the informants to continuously take care of their teeth.

*(I2) ‘It's better to prevent than to keep trying to patch and repair later’.*

They also conveyed feelings of luck, gratitude, satisfaction, pride, and uniqueness due to their caries-free status.

*(I10) “You are kind of proud that you have managed to stay caries-free for 45 years*,* few people have done that.”*

Finally, when receiving favorable feedback from dental professionals regarding their success in maintaining a caries-free condition, the subjects expressed a desire to continue this positive trend into the future.

*(I10) “I get credit every time I’m there*,* so I’m happy with that.”*

*(I1) “What I hope for is that I will remain caries-free into old age*,* FOREVER!”*

## Discussion

This theory-generating study investigated the experiences of salutogenic factors among caries-free adults. The informants attributed their long-term caries-free status to their personal characteristics, attitudes and strategies, as well as various information, influence and support mechanisms from other individuals, dental professionals, schools and society at large. It is theorized that their caries-free status is the result of a combination of several salutogenic factors. These mechanisms can be challenging for both patients and dental professionals to perceive and fully comprehend. Therefore, the informants expressed difficulty understanding why they remained caries free even though they were able to describe the factors they believed contributed to this outcome.

The main theme ‘Remaining caries-free – a personal concern’ reflects the value the informants placed on good oral health, which reflected their view of general health as well as their desire to stay natural and fresh. They described the use of a consequentialist approach as the reason they could influence and take responsibility for remaining caries-free. They also experienced a sense of being unique and felt proud of themselves for remaining caries-free.

The informants emphasized the early influence and involvement of their families as crucial. They described a family environment in which there was an opportunity to discuss personal experiences related to oral health or challenges. This reflects a broader transmission of knowledge, behaviors, and values within the family, indicating that they were raised in supportive homes with caring parents who instilled a strong sense of responsibility. The role of the family with respect to good oral health was also noted in studies by Shmarina et al. [23], who focused on elderly individuals, and Nordström et al. [10], who focused on a young population with low socioeconomic status.

Later in life, it is not only the family that influences the individual to remain caries-free but also one’s extended social network, such as friends, public dental services, schools, and society. Social support in the form of free dental care for school children has been gradually introduced in Sweden since 1938 [24]. The informants highlighted the long-term impact of supportive and empathetic dental staff in the public dental care system. In a study on caries-related behavioral change, Alvenfors et al. [25] reported that in addition to knowledge and skills, the establishment of a strong alliance between staff and patients is key. The way in which information and care relationships are perceived, plays a critical role in shaping patient outcomes [25].

A further crucial contextual factor for this specific age group is the fluoride rinse program, which uses schools as the setting. From the mid-1960s to the late 1980s, schoolchildren were provided with the opportunity to rinse with 0.2% NaF solution on a biweekly basis, coinciding with the school semester [26]. The respondents highlighted this opportunity as important for remaining caries-free even though some of their friends had refrained and did not like the rinses. The informants believed that the dental nurse, also known as the ‘fluoride aunt’, had a major impact on the possibility of remaining caries-free through information and preventive care. The informants were observed to be confident and stable individuals and not influenced by their peers’ attitudes toward fluoride rinses. They were perceived as individuals who adhere to their moral values and not easily swayed by peer pressure.

Health-related advice given by Swedish official authorities may also have contributed to the informants being caries-free. In Sweden, there is generally high confidence and trust in the information given by authorities [27]. The caries-free subjects in this study claimed that they had used information from authorities as a part of their strategy to remain caries-free.

Additionally, the informants identified themselves as meticulous and with a strong desire to maintain optimal health and fulfill the necessary health-related obligations. The informants demonstrated a high level of awareness regarding the sources of information and exhibited a profound understanding of the subject matter pertaining to dental caries. Additionally, they appeared to possess a curiosity that facilitated receptivity to and pursuit of knowledge. Adequate knowledge and comprehension of a condition are prerequisites for active prevention measures. This concept is analogous to the notion of health literacy which may be considered as one determinant for health [28, 29].

One intriguing aspect highlighted by the participants was the perceived impact of genetics. The participants expressed a belief in being born with ‘strong teeth’, a notion that is also supported by the findings of previous studies [10, 23] This belief is substantiated by recent research findings that confirm that genetics does indeed play a role in caries susceptibility [30].

The regular daily use of fluoridated toothpaste and other fluoridated products has been highlighted as crucial, although it has not been frequently discussed from the perspective of the elderly [23]. Over time, the increased availability of fluoridated oral care products has increased the potential for improving oral health and preventing caries. Notably, previous generations did not have access to fluoridated oral hygiene products from a young age, which may have influenced their perspectives to a lesser extent. In contemporary Sweden, national guidelines advocate the utilization of fluoride toothpaste (applied morning and evening without water rinsing) as a fundamental prophylaxis for all individuals, including children and adults [31]. However, even prior to the formulation of these guidelines, the aforementioned recommendations had been utilized by dental care professionals for several decades.

In summary, the informants describe how they believe the factors that have kept them caries-free are related to their individual characteristics and the support they have received from different levels of society throughout their lives. This is similar to the concept of social determinants of health, illustrated by Dahlgren and Whitehead’s rainbow model, which encompasses several successive influencing factors – from individual lifestyle factors to broader socioeconomic, cultural, and environmental conditions [32].

Although the inclusion criterion was a DFT of 0, it is unclear whether the informants were free of initial caries lesions. As initial lesions are not always recorded, it would have been necessary to request dental records and radiographs, which involves increased ethical judgment and was not considered necessary. The choice to exclude M (missing teeth) was made to include caries-free adults who had undergone orthodontic treatment preceded by extractions. The probability that an individual would have had teeth extracted due to caries, yet still be caries-free and without fillings, was assessed as very low. Furthermore, the caries status of their primary dentition is unknown although we asked the informants if they had any memories of having caries lesions in the past. However, the fact that the informants had been free from manifest lesions between 30 and 50 years gives the present study high credibility. Another limitation of this qualitative study is that it analyzed the informants’ perceptions of salutogenic factors contributing to remaining caries-free, which involves both the interpretation and abstraction of collected data. There is a balance to be maintained between the perspectives of the researchers and the informants, requiring careful handling of the researchers’ preunderstandings to remain open to the voices of the informants. Prior to the study, each researcher reflected on the phenomenon and considered how it might impact the interview process and analysis of the interviews. Nevertheless, it should be recognized that the data ultimately represent a collaborative effort between researchers and informants. Providing citations helps reference the original data and strengthens the findings. Additionally, having multiple researchers with diverse backgrounds enhances the data analysis process [33]. However, it is important to be aware that qualitative research of this kind is never completely objective nor reproducible.

It is important to distinguish between three conceptually distinct methodologies or frameworks. Research into risk factors operates within a pathogenic paradigm, identifying factors that increase likelihood for disease. Salutogenesis, by contrast, asks what enables some individuals to maintain health despite exposure to the same risks as others. This proactive, resource-oriented perspective is centered on the origins of health rather than disease [34]. Coping is primarily reactive and describes how individuals deal with poor health when it arises. It is one of the subcomponents of Antonovsky’s salutogenic model through GRR (Generalized Resistant Resources) [34, 35].

Applying the salutogenic model as an overarching framework for caries-free adults is both theoretically justified and uniquely valuable. There are qualitative studies on coping mechanisms or resilience in oral health, but none have used a salutogenic perspective to understand why some people never develop caries. We argue that health literacy, self-efficacy, and sense of coherence together may constitute a salutogenic profile that warrants further investigation as a complementary dimension of caries prevention measures.

A strength of the study was that a strategic and theoretical selection of informants was employed, inspired by grounded theory, which enhanced the trustworthiness of the study [36]. The main advantage of qualitative research is the ability of the methods to discover and generate new knowledge about something that little or nothing is known rather than confirming already existing knowledge. Thus, a qualitative research method, in contrast to quantitative methods, involves an inductive process where the result, on the basis of the collected data, generates new theories or hypotheses [16].

In addition, the results of the study can be interpreted as an illustration of the informants’ reflections on their caries-free status, as well as a flowchart of the various influencing factors that have had a positive impact on remaining caries-free for a significant period of time. This knowledge forms a theoretical basis of influencing factors and strategies for remaining caries-free throughout life. Further studies that explore the findings of the present study via quantified methodologies and use caries-active subjects as controls are needed. A follow-up survey study is planned by the authors, investigating these findings in a larger group of caries-free adults with a caries-active control group, using validated instruments measuring health literacy, self-efficacy, and SOC. A clinical study examining biological factors is also planned.

## Conclusions

The study result supports the view that it is what individuals do at home and in life that enables and maintain a caries free status in individuals, not what dental caregivers do to treat the consequences of the caries disease. This study also highlights the importance of continuity of care with good communication in dental practice. It also underscores the importance of receiving good habits from parents as well as teaching and encouraging a healthy lifestyle at school. This is in line with developing SOC. Being meticulous and responsible as well as experiencing high health literacy may be advantages for remaining caries-free.

## Data Availability

The datasets generated and analyzed during the current studies are not publicly available but can be requested from the corresponding author upon reasonable request.
